# Distinct Fates of Chemokine and Surrogate Molecule Gradients: Consequences for CCR7-Guided Dendritic Cell Migration

**DOI:** 10.3389/fimmu.2022.913366

**Published:** 2022-06-13

**Authors:** Marc Artinger, Oliver J. Gerken, Vladimir Purvanov, Daniel F. Legler

**Affiliations:** ^1^ Biotechnology Institute Thurgau (BITg) at the University of Konstanz, Kreuzlingen, Switzerland; ^2^ Graduate School for Cellular and Biomedical Sciences, University of Bern, Bern, Switzerland; ^3^ Faculty of Biology, University of Konstanz, Konstanz, Germany; ^4^ Theodor Kocher Institute, University of Bern, Bern, Switzerland

**Keywords:** chemokine gradient formation and maintenance, CCL19, CCL21, CCR7, dendritic cell migration, fluorescent chemokines

## Abstract

Chemokine-guided leukocyte migration is a hallmark of the immune system to cope with invading pathogens. Intruder confronted dendritic cells (DCs) induce the expression of the chemokine receptor CCR7, which enables them to sense and migrate along chemokine gradients to home to draining lymph nodes, where they launch an adaptive immune response. Chemokine-mediated DC migration is recapitulated and intensively studied in 3D matrix migration chambers. A major caveat in the field is that chemokine gradient formation and maintenance in such 3D environments is generally not assessed. Instead, fluorescent probes, mostly labelled dextran, are used as surrogate molecules, thereby neglecting important electrochemical properties of the chemokines. Here, we used site-specifically, fluorescently labelled CCL19 and CCL21 to study the establishment and shape of the chemokine gradients over time in the 3D collagen matrix. We demonstrate that CCL19 and particularly CCL21 establish stable, but short-distance spanning gradients with an exponential decay-like shape. By contrast, dextran with its neutral surface charge forms a nearly linear gradient across the entire matrix. We show that the charged C-terminal tail of CCL21, known to interact with extracellular matrix proteins, is determinant for shaping the chemokine gradient. Importantly, DCs sense differences in the shape of CCL19 and CCL21 gradients, resulting in distinct spatial migratory responses.

## Introduction

Directed cell migration is a fundamental process that controls many physiological functions, including protective immunity (1). Immune cell migration is governed by the cell’s interaction with substrates of the environment and is orchestrated by extracellular directional guidance cues established primarily by members of the chemokine family (2, 3). As chemokines were originally described as secreted chemotactic cytokines, the paradigm of ‘chemotaxis’ emerged, in which cells migrate along a soluble, diffusion-based gradient towards the source of the chemoattractant (4). However, locally produced chemokines interact with extracellular matrix proteins of the surrounding environment (5), which prevents free diffusion and facilitate confined chemokine gradient formation (6–9). Importantly, negatively charged amino acid residues located typically at the C-terminal tail of chemokines control the interaction with extracellular substrates and likely gradient formation. However, information on chemokine distribution and quantitative assessments of chemokine gradients remain rare.

One of the best-studied chemokine gradient is that of CCL21 (8, 10). In the dermis, CCL21 is produced and secreted by lymphatic endothelial cells, kept locally through electrostatic interactions with the environment, and forms a steeply decaying gradient into the interstitial tissue (8). Pathogen experienced DCs are able to sense and migrate along this local CCL21 gradient in a CCR7 dependent manner to enter the lymph vessel on their way to the draining lymph node (8). Within the lymph node, CCL19, the second ligand for CCR7, seems to predominantly control DC migration and positioning (11). Notably, haptotactic DC migration along a substrate-bound CCL21 gradient and chemotactic, CCL19-mediated migration can be recapitulated in fabricated 3D collagen migration devices (12–14). However, which of the two CCR7 ligands is more potent in attracting DCs is controversial (13–16).

A major caveat in the field is that chemokine gradient formation and maintenance in 3D migration devices is generally not determined. Instead, fluorescent probes with comparable molecular weights to chemokines, mostly labelled dextran, are used as surrogate molecules (14, 17–19), thereby ignoring the electrostatic characteristics of chemokines. Importantly, the polar C terminal tail of CCL21 is well known to interact with extracellular matrix proteins, including collagen (20, 21). Hence, in the present study we set out to measure chemokine gradient formation and maintenance in time and space of a 3D collagen migration device. To do so, we used our recently developed site-specifically, fluorescently labelled CCL19 and CCL21 (22) in a side-by-site comparison with the commonly used surrogate molecule dextran.

## Material and Methods

### Production of Fluorescently Labelled Chemokines

Cloning and production of S6-tagged human CCL19 and CCL21 have been described previously (22). Human S6-tagged CCL21trunc, corresponding to amino acids 1-79 of the mature chemokine (23), was generated accordingly. Recombinant S6-tagged chemokines were site-specifically, enzymatically labelled using the phosphopantetheinyl-transferase Sfp with the fluorescent dye Dy549P1 (Dyomics, Jena, Germany) according to the established protocol and purified *via* C18 reverse phase HPLC (22).

### Isolation of Human Monocyte-Derived Dendritic Cells (MoDCs)

Primary human monocytes were isolated from the blood of healthy donors, differentiated into MoDCs in GM-CSF/IL-4 and matured with a cytokine cocktail (TNFα/IL-6/IL-1β/PGE_2_) using standard protocols (24). Individual donors gave written consent and donations were approved by the local ethics committee.

### Monitoring of Chemokine Gradient Formation and MoDC Migration in 3D Matrix

Gradient formation of fluorescent chemokines and subsequent migration of matured human MoDCs within a 3D matrix was performed in Ibidi µ-slide chambers (Ibidi, Martinsried, Germany). Briefly, matured MoDCs were collected in RPMI 1640 medium supplemented with 10 % FCS and 1 % penicillin/streptomycin at a concentration of 10^7^ cells/ml. For collagen polymerization, 20 μl 10× DMEM, 10 μL 7.5 % NaHCO_3_, and 150 μL PureCol collagen I (Advanced Biomatrix, Carlsbad, CA, USA) were carefully mixed with 90 μL cells. The cell-collagen mixture was mounted into µ-slides according to the manufacturer’s instructions. Tetramethylrhodamine conjugated dextran of 10 kDa (Invitrogen, ThermoFisher), or fluorescent chemokines, all at a concentration of 100 nM, were applied to the right reservoir, whereas DMEM without phenol red (Gibco, ThermoFisher) was added to the left reservoir of the Ibidi-chamber. DC migration and gradient formation was monitored and recorded by time-lapse bright field and fluorescence microscopy for 3 or 5 hours on a Axiovert 200M (Zeiss, Oberkochen, Germany) equipped with a Tokai Hit INU (Tokai, Shizuoka, Japan) incubation system acquiring images every 5 minutes.

Cellular 3D migration was evaluated using the ‘chemotaxis and migration tool’ (Ibidi) to determine the velocity and forward migration index. To assess gradient formation, images were analyzed with FIJI. Images were cropped to the region of interest represented by the collagen matrix to exclude the reservoirs. The distribution of mean fluorescent intensities was evaluated using the rectangular intensity histogram tool covering the entire matrix. Individual data sets were evaluated in Prism 9.0 (Graphpad, La Jolla, CA, USA) applying the one-phase decay model.

## Results

### Visualization and Quantification of Dextran, CCL19 and CCL21 Gradient Formation and Maintenance

**Figure 1:**
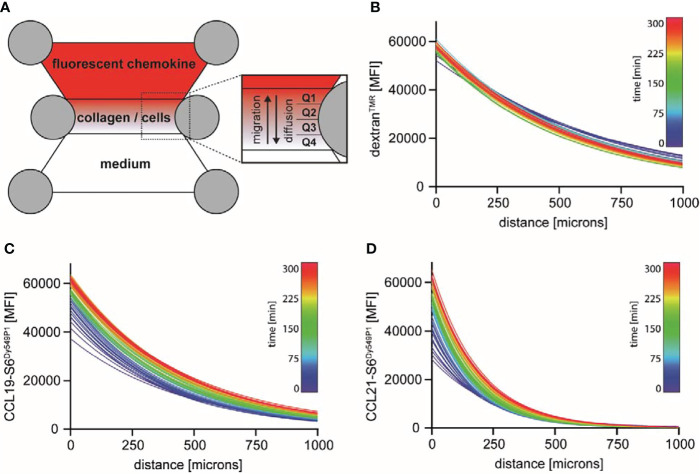
Visualization and characterization of fluorescently labelled dextran, CCL19 and CCL21 gradients in 3D migration chambers. **(A)** Schematic representation of a 3D migration chamber. Cells are embedded in a defined 3D collagen I matrix of an Ibidi μ-slide suitable for time-lapse fluorescence microscopy. Fluorescently labelled molecules and control medium are filled into the adjacent connected reservoirs, such that a gradient is established over time following a source-and-sink principle. The central chamber corresponds to the observation area, which we subdivided into four quarters (Q1-Q4) for certain analysis. **(B-D)** Distribution of fluorescently labelled molecules in the central observation chamber was measured by time-lapse fluorescent microscopy. One reservoir was filled with 100 nM dextran^TMR^ **(B)**, CCL19-S6^Dy549P1^ **(C)** or CCL21-S6^Dy549P1^ **(D)**. Mean fluorescence intensity (MFI) of the labelled proteins within the 3D matrix was monitored over time for 5 hours, fitted with a one-phase decay model and color graded from blue (t = 0min) to red (t = 300min). One out of two independent experiments is shown.

Chemokine-guided leukocyte migration in a 3D environment is nowadays routinely monitored in standardized, commercially available devices. In such a device, e.g. the Ibidi µ-slide, cells are embedded in a defined gel matrix within an enclosed central chamber. The two connected adjacent reservoirs are filled with either a chemoattractant or medium that diffuse into the central gel following a source-to-sink model. Gel-embedded cells orient and migrate in 3D towards the higher concentration of the chemoattractant (**Figure 1A**). Due to the lack of tools to visualize chemokines in these 3D migration chambers, gradient formation has often been taken for granted or eventually estimated by the use of fluorescently labelled surrogate molecules. The most commonly used surrogate molecule is dextran with a molecular weight of 10 kDa to roughly match the size of a chemokine. We used tetramethylrhodamine-labelled dextran of 10 kDa (dextran^TMR^), applied it into one reservoir of the migration chamber and measured its diffusion into the 3D collagen I matrix over time by time-lapse fluorescence microscopy. Plotting the diffusion profiles of the dextran^TMR^ intensity at the centered longitudinal axis of the gel chamber as a function of the distance to the reservoir for increasing time points revealed a stable, nearly linear gradient across the entire matrix chamber (which is 1000 microns) with only minor changes in the fluorescence distribution over the whole period of measurement, i.e. five hours (**Figure 1B**). Precise mapping of site-specifically labelled and fully functional CCL19-S6^Dy549P1^ (22) in the 3D collagen I matrix directly after its application to the reservoir revealed a high fluorescent signal near the reservoir edge, which rapidly decreased over the first hundred microns of the gel chamber (**Figure 1C**). After five hours of incubation, the distribution of CCL19-S6^Dy549P1^ within the 3D matrix converged to the one observed for dextran^TMR^. Notably, the CCL19-S6^Dy549P1^ chemokine gradient stretched across the entire matrix over the full period of measurement (**Figure 1C**). By contrast, fluorescently labelled CCL21-S6^Dy549P1^ (22) sparsely entered deep into the collagen matrix within the first 30 minutes and the fluorescence intensity derived from CCL21-S6^Dy549P1^ steeply decreased with the distance to the reservoir (**Figure 1D**). Notably and in marked contrast to dextran^TMR^ and CCL19-S6^Dy549P1^, the high-slope exponential decay of the CCL21-S6^Dy549P1^ gradient was only measurable over a few hundred microns into the collagen matrix and remained undetectable at the opposite end of the chamber (**Figure 1D**). Such a steep and short-distance spanning CCL21 gradient shape has also been observed in the dermal interstitium (8). Although no *in vivo* data on a CCL19 gradient shape has been reported yet, our quantitative assessments of chemokine gradients in a 3D environment reveal distinct shapes for gradients with different chemokines, and importantly, uncovers the limitation of dextran as general surrogate marker for monitoring chemokine gradients.

### The Charged C-Terminus Is Determinant for Shaping the CCL21 Gradient

**Figure 2:**
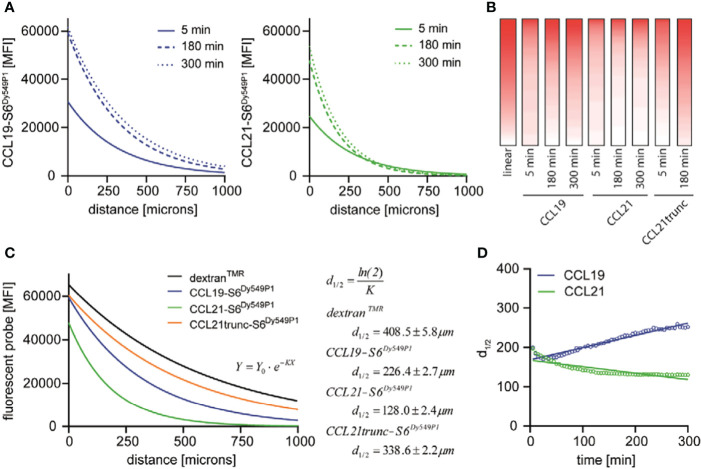
CCL19, CCL21 and CCL21trunc establish stable gradients of distinct shapes. **(A)** Mean fluorescence intensity (MFI) of CCL19-S6^Dy549P1^ and CCL21-S6^Dy649P1^ in relation to the distance to the reservoir measured after 5 min, 180 min and 300 min of incubation. **(B)** MFI values of CCL19-S6^Dy549P1^, CCL21-S6^Dy649P1^ [taken from **(A)**] and CCL21trunc-S6^Dy549P1^ were translated in a pseudocolor representation, and supplemented with an artificial linear gradient for comparison. **(C)** Applying a one-phase exponential decay function reveals different 50 % initial fluorescence values (d_1/2_) for dextran^TMR^, CCL19-S6^Dy549P1^, CCL21-S6^Dy549P1^ and CCL21trunc-S6^Dy549P1^ after 180 min. **(D)** Spatial distribution of d_1/2_ as the ‘mean chemokine diffusion’ over time increases for CCL19-S6^Dy549P1^, but not for CCL21-S6^Dy549P1^.

Our data indicate a time-dependent formation and shaping of the chemokine gradients. This poses the question of how long it takes the chemokines to establish stable gradients within the 3D collagen matrix. Both fluorescent chemokines rapidly (in the time range of minutes) establish well-defined gradients within the 3D collagen matrix, although the steepness and width of the gradients adjust over time, until quasi-stable gradients are attained after 180 minutes that do not substantially change any longer (**Figures 2A, B**). The exponential decay, particularly of the CCL21-S6^Dy549P1^ gradient, clearly indicates that the chemokine cannot freely diffuse within the 3D collagen matrix like its surrogate dextran^TMR^. The steeper gradient of CCL21-S6^Dy549P1^ is likely to have its nature in the polar C-terminal tail, which is known to electrostatically interact with collagen and other extracellular matrix proteins (20, 21). To substantiate this, we fluorescently labelled a naturally occurring CCL21 variant [amino acids 1-79 of the mature CCL21 peptide (23)], referred to as CCL21trunc-S6^Dy549P1^ which lacks the charged C-terminal tail. Indeed, CCL21trunc-S6^Dy549P1^ showed a close to linear distribution within the 3D collagen matrix (**Figure 2B**).

The shape of the chemokine gradients could best be fitted using an exponential one-phase decay function of the type *Y* = *Y*_0_ · *e* ^–^ *^KX^* after subtracting the background fluorescence by setting the plateau equal to zero. Here, *Y*_0_ describes the initial fluorescence, whereas *K* is the rate constant expressed in reciprocal of the X-axis units. Based on the calculation of *K*, the chemokine half-life corresponds to 50 % of the initial fluorescence derived by $\frac{ln\left(2\right)}{K}$ , resulting in d_1/2_. From this fit, d_1/2_ of CCL21-S6^Dy549P1^ was the shortest after three hours of gradient formation (d_1/2_ = 128.0 ± 2.4 μm) and remained relatively stable over time (**Figure 2C**). However, CCL19-S6^Dy549P1^ showed a more distant drop in fluorescence intensity (d_1/2_ = 226.4 ± 2.7 μm) (**Figure 2C**), whereas CCL21trunc-S6^Dy549P1^ and dextran^TMR^ showed a comparable distribution among the gradient with comparable 50 % fluorescence intensities (d_1/2_ = 338.6 ± 2.2 μm for CCL21trunc-S6^Dy549P1^ and d_1/2_ = 408.5 ± 5.8 μm for dextran^TMR^, respectively) (**Figure 2C**). Moreover, calculating the chemokine specific diffusion d_1/2_ revealed a slight temporal decrease for CCL21-S6^Dy549P1^ as the initial fluorescence within the first chamber quarter raises over time and subsequently lowers the spatial localization of d_1/2_ (**Figure 2D**). By contrast, the spatiotemporal distribution of d_1/2_ for CCL19-S6^Dy549P1^ follows an ascendant line based on the forward shift of the 50 % fluorescence intensity caused by free chemokine diffusion underlining chemotactic gradient formation (**Figure 2D**).

### DCs Sense Differences in the CCL19 and CCL21 Gradients Resulting in Distinct Migratory Responses

**Figure 3:**
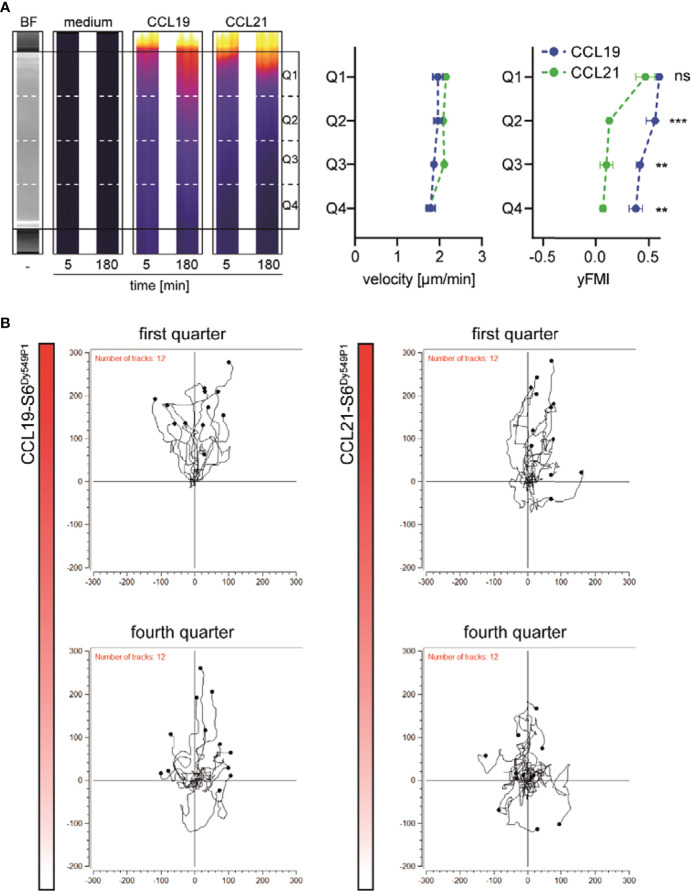
Distinct shapes of CCL19 and CCL21 gradients translate into different migratory responses of human DCs. **(A)** Representative cropped microscopic images of the central 3D migration chamber. Bright field (BF) as well as pseudocolored fluorescence images of medium, CCL19-S6^Dy549P1^ and CCL21-S6^Dy549P1^ at indicated time points derived from one out of three independent experiments are shown (left panel). Velocity and forward migration index in the y-axis (yFMI) of human matured MoDCs migrating along the chemokine gradients in each quarter are shown in the right panel. Mean values ± SD for n = 3, statistical analysis: two-way ANOVA with Sidak post-test. **(B)** Spider plots of individual tracks of migrating MoDCs in quarters Q1 and Q4.

To investigate functional consequences for guiding DCs by the different shapes of the chemokine gradients, we embedded human matured MoDCs into the central 3D collagen matrix and let them migrate along the CCL19-S6^Dy549P1^ or CCL21-S6^Dy549P1^ gradient. We subdivided the migration chamber into four consecutive quarters, Q1 to Q4, of which Q1 is the one closest to the chemokine reservoir (**Figure 3A**). Cells in each quarter that moved more than a cell diameter were tracked to determine the velocity and the forward migration index (yFMI) of the migrating MoDCs. Notably, MoDCs that started in Q1 migrated with a comparable, statistically indistinguishable velocity and yFMI in either of the two chemokine gradients (**Figure 3A**). Moreover, all MoDCs sensed and migrated towards the chemokine source (**Figure 3B**). Intriguingly, a similar velocity of migrating MoDCs was observed in all four quarters for the two CCR7 ligands (**Figure 3A**). By contrast, MoDCs starting in Q2 of the CCL21-S6^Dy549P1^ gradient, although still motile, show a significantly reduced directional migration behavior as manifested with a significantly lower yFMI (**Figure 3A**). Notably, MoDCs from all quarters were able to sense and follow the CCL19-S6^Dy549P1^ gradient, whereas MoDCs in Q4 were motile but failed to sense and migrate along the CCL21-S6^Dy549P1^ gradient (**Figure 3B**).

## Discussion

Cell migration along chemokine gradients is a fundamental process not only during development but also in orchestrating protective immune responses. Major advances have been achieved in the design and fabrication of sophisticated devices to study cell migration dynamics in 3D environments in real-time. By contrast, how chemokines distribute in such devices and how gradients are formed and maintained is sparsely investigated. The main reason for the poor information is the lack of appropriate tools, i.e. the availability of fluorescently labelled chemokines. Fluorescent surrogate molecules like fluorescently labelled dextran of the molecular weight of chemokines have been used instead (14, 17–19). Therefore, we recently took on this problem and have designed and produced a first and a second generation of fluorescently labelled chemokines (22, 25).

In the present study, we now show the limitations of such surrogates in a defined 3D matrix. We demonstrate that the surrogate dextran, which owns non-charged surface characteristics, essentially follows a near free, linear diffusion over time within the 3D matrix. In marked contrast, chemokines possess clusters of charged amino acids exposed at their surface, and hence establish exponential-like gradients in the 3D collagen migration chamber. Indeed, CCL21 with its extended, highly polar C-terminus formed the steepest gradient. This goes along with its profound interaction with glycosaminoglycan structures and other extracellular substrate components that is abrogated in CCL21trunc (20, 21). Importantly, electrostatic interactions between the chemokine and the extracellular matrix substrate restrain the free diffusion of the chemokine, such that chemokine gradients are of much shorter distance than surrogate dextran gradients. Consequently, these particular chemokine properties must be taken into consideration when cell migration along chemokine gradients is investigated. In fact, the distance from the chemokine source and the shape of the chemokine gradient is decisive whether a cell is able to sense and follow the guidance cue or not. Consistent with a previous study on murine chemokines, we found that a stable gradient was established after about two to three hours for CCL19-S6^Dy549P1^ and CCL21-S6^Dy549P1^, whereas gradient formation of dextran^FITC^ was not temporally affected (13). The steep gradient shape of CCL21-S6^Dy549P1^ in our 3D matrix mimics the fast decay of the murine CCL21 gradient away from the lymphatic vessels reported in the dermis (8, 26). We further show that gradients formed by two ligands for CCR7 are of a different shape and point to a limited meaningfulness of using dextran as surrogate molecule to characterize chemokine gradients.

## Data Availability Statement

Datasets for this study are deposited on Zenodo and are publicly available under a Creative Commons Attribution 4.0 International license, doi: 10.5281/zenodo.6362275. The data can be found at https://zenodo.org/record/6362275#.Yp81SKjMIuU.

## Ethics Statement

The studies involving human participants were reviewed and approved by Ethics Committee of the University of Konstanz. The patients/participants provided their written informed consent to participate in this study.

## Author Contributions

Conceptualization, MA and DL. Methodology, MA, OG, and VP. Investigation, MA, OG, and VP. Visualization, MA, and DL. Writing - original draft, MA and DL. Writing - review and editing, all authors. Supervision, DL. Project administration, DL. Funding acquisition, DL. All authors contributed to the article and approved the submitted version.

## Funding

This research was supported in parts by research funding from the Swiss National Science Foundation, grant number 310030_189144, the Thurgauische Stiftung für Wissenschaft und Forschung, and the State Secretariat for Education, Research and Innovation.

## Conflict of Interest

The authors declare that the research was conducted in the absence of any commercial or financial relationships that could be construed as a potential conflict of interest.

## Publisher’s Note

All claims expressed in this article are solely those of the authors and do not necessarily represent those of their affiliated organizations, or those of the publisher, the editors and the reviewers. Any product that may be evaluated in this article, or claim that may be made by its manufacturer, is not guaranteed or endorsed by the publisher.
